# Digital Disability: A New Risk to Older People in Digital Societies

**DOI:** 10.3389/ijph.2024.1607303

**Published:** 2024-06-28

**Authors:** Kai Zhang

**Affiliations:** School of Public Administration, Sichuan University, Chengdu, China

**Keywords:** digital disability, older people, digital technology, digital inclusion, challenge

**The IJPH series “Young Researcher Editorial” is a training project of the Swiss School of Public Health.**

There are now more than 761 million people over 65 years old [1], and the proportion of older people will continue to increase, reaching 1.6 billion by 2050 [2]. Older people often find it difficult to adapt to digital technology, which can disadvantage them in an increasingly digital society [3, 4]. Today, digital devices and services are used to access everything from social groups to healthcare [5], and barriers to using digital tools such as smartphones and platforms such as social media can reduce the access of older people to healthcare services like healthcare. Many healthcare providers now expect clients to visit digital platforms to, e.g., schedule appointments and access medical records, but older people may not be able to navigate these digital healthcare systems as easily as younger people. These difficulties can be described as a “digital disability” [6] that can marginalize older people because they lack access to information, social interaction, health services, and digital inclusion [7]; marginalization can, in turn, increase mental health risks or social discrimination [8].

A “digital disability” is the aggregated technical, psychological, and cultural disabilities that accrue when an individual lacks the skills to function in digital society. Not all older people are digitally disabled, and some older people function well in the absence of digital technology, so when we measure functionality in digital environments, we must look at how an individual’s behavior is influenced by digital trends within a specific socioeconomic context [9]. Digital disability is distinguished from the “digital divide,” which focuses on unequal access to and distribution of digital technologies, and from “digital exclusion,” which emphasizes a person’s subjective experience and the conseqences of their exclusion from digital society. Instead, digital disability refers to a person’s loss of autonomy and control in a digital society, such as being unable to independently access essential online services. Though many older people suffer from digital disability, there are other digitally disadvantaged groups, including disabled people, poor people, and people with low education.

To help digitally disadvantaged people fully participate our digital society, we must understand why digital disability develops and then devise strategies for preventing it. First, we must conduct research and administer surveys to identify specific barriers, e.g., lack of familiarity with technology, cognitive decline, or physical limitations. Second, we must develop targeted education and training programs to address the specific needs and challenges of people with digital disability. These programs should build digital literacy skills, provide step-by-step guidance on using digital devices and services, and support people with digital disabilities in overcoming common obstacles. Third, we must design and develop accessible and user-friendly digital products and services with this user group in mind, e.g., implement intuitive interfaces, adjustable font sizes, clear navigation, and voice command options that accommodate users with varying levels of digital proficiency and physical abilities. Fourth, we must create opportunities for people with digital disabilities to engage with digital technology in social and community settings through, e.g., group training sessions, online forums, and targeted social media groups. We must also create intergenerational learning environments that reduce isolation and encourage peer support. Fifth, we must retain and provide non-digital access to central services (e.g., public transport, healthcare, living expenses, etc.) so that people with digital disability can access essential healthcare and support services.

Proactive measures by governments and older people are essential to promote digital inclusion, empowerment, and lifelong learning to address digital disability. This will not only give individuals more independence and have better access to digital services, but will also build a more inclusive and resilient digital society. Active inclusion in the digital society is essential to the health of older persons and to ensure that they share in their digital wellbeing.
